# Plant health in the era of global changes, holobiont biology, and microbiome-based solutions

**DOI:** 10.1093/hr/uhaf364

**Published:** 2026-01-02

**Authors:** Edda Francomano, Meriem Miyassa Aci, Saveria Mosca, Nesma Zakaria Mohamed, Giovanni Enrico Agosteo, Maria Giulia Li Destri Nicosia, Antonino Malacrinò, Leonardo Schena

**Affiliations:** Department of Agriculture, Università degli Studi Mediterranea di Reggio Calabria, Reggio Calabria, Italy; Department of Agriculture, Università degli Studi Mediterranea di Reggio Calabria, Reggio Calabria, Italy; Department of Agriculture, Università degli Studi Mediterranea di Reggio Calabria, Reggio Calabria, Italy; Department of Agriculture, Università degli Studi Mediterranea di Reggio Calabria, Reggio Calabria, Italy; Agronomy Department, Faculty of Agriculture, Cairo University, Giza, Egypt; Department of Agriculture, Università degli Studi Mediterranea di Reggio Calabria, Reggio Calabria, Italy; Department of Agriculture, Università degli Studi Mediterranea di Reggio Calabria, Reggio Calabria, Italy; Department of Agriculture, Università degli Studi Mediterranea di Reggio Calabria, Reggio Calabria, Italy; Department of Biological Sciences, Clemson University, Clemson, SC, USA; Department of Agriculture, Università degli Studi Mediterranea di Reggio Calabria, Reggio Calabria, Italy

## Abstract

Agriculture faces unprecedented challenges due to climate change, increasing food demand, and resource scarcity, which needs sustainable and innovative solutions. This review explores the emerging paradigm of holobiont biology (host and its microbiome as biological unit) in the context of emerging plant health challenges driven by global changes. We highlight three critical challenges: the rise of complex plant syndromes, the emergence and re-emergence of plant diseases, and the consequences of dysbiotic plant microbiomes. We discuss how microbiome-based strategies can enhance plant resilience, reduce reliance on agrochemicals, and foster sustainable agriculture. Integrating these strategies with advanced frameworks, such as holo-omics and machine learning, opens avenues for microbiome-based solutions to address agricultural challenges in the era of global changes, ensuring resilient crop systems and planetary health.

## Introduction

Agriculture is facing the major challenge of feeding an exponentially increasing global population while guaranteeing planetary health and facing the consequences of global changes [1–3]. At the same time, global food demand rises, the global availability of resources (e.g. arable land, groundwater, nutrients) is decreasing [4–6]. Compared to 2010, by 2050, we will witness a 1 to 2 billion increase in global population size [3] and an increase in food demand of 35% to 56% [7]. However, up to 40% of agricultural production is currently lost due to pests and pathogens [8], and ~ 24% of production is lost throughout the supply chain and never reaches consumers [9]. Thus, while we need more food, a wide slice of it is lost in the field or throughout the supply chain, and we are at risk of losing the arable land to support the forecasted production. It is now more clear than ever that traditional agricultural practices are not suitable for the agriculture of the future.

Global changes are directly affecting agricultural production [10] and are amplifying the environmental impact of agriculture [11]. The variations in the abiotic environment consequential to global changes can have profound influences on the interactions between plants and their pathogens. Indeed, weather anomalies can influence both disease outbreak in agricultural settings and disease pressure in natural plant communities [12]. In addition, several recent reports show that pathogens once considered of marginal importance can emerge, or re-emerge, as important threats to crops due to the increasing climatic variability [13, 14], as a consequence of the host or geographical range expansion, or a combination of both. Similarly, microorganisms that were not considered a threat to plant health, under new climatic conditions, can become agents of complex plant syndromes resulting from the combined action of abiotic stressors and the attack of one or more pathogens [15]. In a wider scenario, changes in environmental conditions can also disrupt the beneficial interactions between host plants and their associated microbial communities, with severe detrimental effects to plant health [16, 17]. Solving future challenges in plant protection, thus, requires going beyond the ‘one pathogen–one disease’ paradigm to look at the whole picture of plant–microbiome–environment interactions [18]. Indeed, understanding the mechanisms regulating interactions between plants and their microbiomes offers opportunities to harness microbiome-based solutions to solve novel challenges for plant and planetary health [19].

One lesson we have learned during the past decade is that planetary health is tightly linked to the structure and functioning of the microbial communities inhabiting all the ecological niches in our planet, including those associated with animals and plants [20]. We often think of plants and pathogens as separate units, although they are part of a complex network of interactions between the host plant and all the microbial communities inhabiting the different plant organs. These microbiomes host prokaryotic and eukaryotic communities, together with viruses and extrachromosomal genetic elements, that are tightly linked to host biology, ecology, evolution, and health so that hosts and their microbiomes are actually a single biological unit: the holobiont [21–23]. The concept of holobiont challenges the idea of siloed components of host biology, but it is often overlooked in plant protection, although both the host plant and its associated microbial communities, in concert, are gatekeepers of plant health. Global changes threaten the stability and function of the holobiont [24–26], and this can have cascading negative consequences on planetary health [21]. Indeed, global changes impact both the host and its microbiome, posing new challenges for holobiont health. While plant protection, holobiont biology, and global changes are often treated separately, here, we aim at addressing this knowledge gap by discussing novel challenges for the plant holobiont health driven by global changes, focusing on three major cases that fall outside the ‘one pathogen–one disease’ framework and are directly influenced by global changes: (i) complex plant syndromes, (ii) (re)emerging plant diseases, and (iii) dysbiotic plant microbiomes. We then discuss how microbiome-based solutions and new frameworks and tools can contribute to their solution.

## Global changes and new challenges for the plant holobiont health

Global changes and the resulting increase in climate variability represent a key challenge for the future of crop protection [27]. Changes in the abiotic environment can result in biological consequences for each component within the holobiont (Table 1), which might gain or lose advantage under new conditions, resulting in the dysbiotic alteration of plant–microbe interactions and possible detrimental effects for the host plant [17, 25]. In turn, this can lead, for example, to the (re)emergence of pathogens in new locations or crops [35, 36] or the development of complex diseases and syndromes [15].

**Table 1:** Effect of a selection of global change factors on plant and soil microbiomes, the host plant, the holobiont, and holobiont health.

**Global change factors**	**Plant and soil microbiomes**	**Host plant**	**Holobiont**	**Holobiont health**
**Warming**	Warming reshapes plant microbiomes by reducing diversity (especially beneficial microbes) and increasing pathogen load. These changes increase microbial nutrient uptake, boost some nitrogen-fixers, and increase disease susceptibility [28].	Warming forces plants to adjust their physiology shifting their photosynthetic temperature optimum and reduce respiration. However, plants in already warm climates have limited capacity to acclimate, leading to reduced photosynthesis, growth, and carbon uptake [29].	Warming disrupts the plant holobiont by reshaping microbial community assembly, altering nutrient uptake, stress tolerance, and immune regulation, weakening the plant ability to cope with increasing temperature and other stresses. Because of the tight connection between plant physiology and microbiome composition and function, warming-induced changes can influence plant growth and stress resilience [25].	Warming predisposes plants to decline syndromes by prolonged stress and nutrient limitation. Geographical- and host-range expansion of pathogens and longer conducive seasons promote (re)emerging diseases. Progressive replacement of keystone mutualists by stress-tolerant opportunists increases the probability of dysbiosis.
**Drought**	Drought significantly alters the structure and function of plant-associated microbial communities, typically reducing overall microbial diversity in the rhizosphere and root microbiomes, selecting for drought-tolerant taxa, and enriching specific drought-responsive microorganisms that may support host under stress [30].	Drought leads to reduced water uptake, stomatal closure, decreased photosynthesis, accumulation of reactive oxygen species, and disruption of cellular homeostasis. Plants respond by activating stress signaling pathways, increasing osmoprotectant synthesis (e.g. proline), and triggering hormonal changes to conserve water and maintain metabolic balance. Prolonged drought typically impairs growth, biomass accumulation, nutrient transport, and reproductive success, often resulting in decreased yields, altered development, and greater susceptibility to other stresses [30].	Changes in soil moisture and plant root exudation under drought shift the availability of nutrients and metabolites in the rhizosphere, reshaping microbial colonization and function. These shifts often favor microbes capable of osmoprotection, stress signaling, and metabolic resilience, but can also reduce beneficial symbionts when water deficits become severe [30].	Decline syndromes can emerge from prolonged stress and nutrient limitation. Pathogens can gain a competitive advantage under host stress, facilitating the (re)emergence diseases. Strong and prolonged drought events can reduce microbiome diversity and functional redundance, increasing the likelihood of dysbiosis.
**Increase in atmospheric CO**_**2**_	Microbial respiration, and enzyme activities typically increase under higher CO₂, boosting nutrient mineralization, improving nutrient availability [31, 32].	Elevated CO₂ generally stimulates plant photosynthesis by increasing CO₂ assimilation and reducing stomatal conductance, which can enhance water-use efficiency and boost early growth. However, long-term exposure leads to photosynthetic acclimation which diminishes the initial gains. Elevated CO₂ also alters plant resource allocation, often increasing root growth but decreasing nutrient uptake and translocation [33].	Elevated CO₂ reshapes the plant microbiome by modifying root exudation patterns and altering the resources available to soil microbes, favoring groups such as mycorrhizal fungi, while potentially reducing saprotrophs and influencing nutrient cycling processes [31, 34].	Greater carbohydrate availability may promote pathogen fitness. Carbon-driven dominance of fast-growing taxa can weaken mutualist networks and predispose the holobiont to dysbiosis.

### Dysbiotic plant microbiomes

**Figure 1:**
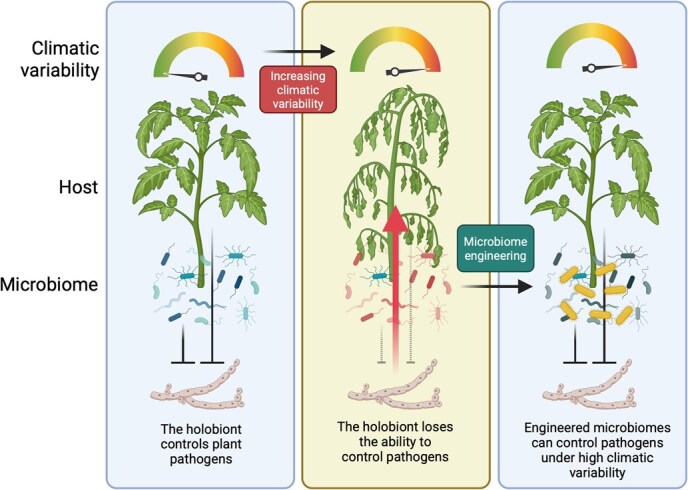
Under low climatic variability, the holobiont is able to counteract pathogens through the influence of both the host and the microbiome components. However, under stresses induced by increasing climatic variability (e.g. warming, drought, elevated CO_2_, see Table 1), the interactions within the holobiont can be altered with consequences on its overall resistance to stressors, thus giving the possibility to pathogens to cause disease. On the other hand, understanding the holobiont can help us to engineer microbiomes to be resilient to increasing climatic variability and protect plants from new health challenges. Created in https://BioRender.com

Global changes do not solely influence plant–pathogen interactions but plant–microbiome interactions more in general [25]. As many of the interactions within the plant holobiont are mostly beneficial, supporting plant nutrition, resilience to abiotic stressors, and resistance to pests and pathogens, the disruption of interactions within the holobiont can lead to detrimental effects for plants [16] and the development of diseases that do not follow the classic paradigms of ‘one pathogen–one disease’. In this context, the host can lose its capacity to regulate its associated microbial communities [16], which can happen as a consequence of external stressors, such as those driven by global changes, leading to a dysbiosis. This loss of microbiome regulation by the host plant can alter the ecological processes behind microbiota recruitment and assembly, impacting diversity, composition, and function (including functional redundancy and complementarity) of plant-associated microbial communities [16], and ultimately reducing the ability of the holobiont to counteract stressors. While it seems that a dysbiotic microbiome does not directly cause the disease, its particular composition can instead facilitate pathogens to infect plant tissues [37–40] or lead to the development of a pathobiome [41, 42], a disease-promoting configuration of the plant microbiome that can cause disease across different plant compartments [16]. Strikingly, some pathogens can induce dysbiosis as part of their pathogenic strategy by modulating the host microbiome and creating niches conducive to the pathogen, as observed in *Xanthomonas* spp. on *Arabidopsis thaliana* [43] and in *Verticillium dahliae* on tomato and cotton [44]. These examples clearly show us how important it is to understand the interactions within the holobiont, as both the host and its microbiome play a role in preventing pathogens from causing diseases. It is thus essential to keep digging into the causes and consequences of microbiome dysbiosis, as maintaining a resilient, healthy plant microbiome is key to preventing pathogens from exploiting the pathobiome and generating diseases. Under increasing climatic variability, the higher incidence of environmental stressors can increase the likelihood that plants can lose their ability to regulate their associated microbial communities. Although these events might be transient, they represent an opportunity for pathogens to avoid the holobiont defenses and produce detrimental effects to plants (Fig. 1). Maintaining beneficial interactions within the holobiont is thus essential to manage new and old threats to plant health.

### Complex plant syndromes

Complex plant diseases, for example, declines and diebacks, are diseases with unclear etiology and often fall outside the one pathogen–one disease paradigm [45]. The Kiwifruit Vine Decline Syndrome (KVDS) is a prime example of plant syndrome with a complex etiology. Early hypotheses pointed toward climate change as a major factor driving the spread of this condition [46–48]. Later studies showed that KVDS has a biotic origin [49]. Although this syndrome has been studied for the past 10 years, it is still unclear if KVDS is induced by one or several agents, and whether these agents have been actually identified or if the answer lies in hypotheses still unexplored [50]. For example, the consequences of climatic changes (e.g. increasing frequency of droughts and floods) and agricultural practices that reduce drainage (e.g. soil compaction, excess of irrigation during summer) can all induce further stress in the plant holobiont and create such conducive conditions that weak pathogens can emerge as a serious threat within orchards. KVDS is an example of plant disease which induction, according to the current knowledge, seems to be directly influenced by global changes and the soil microbiome. Similar events seem to be more commonly observed in forests, and several reports suggest an increasing incidence of tree declines and diebacks following variation in climatic conditions [51]. Examples include the Silver Fir Decline [52], the Araucaria decline [52], the Beech Leaf Disease [53, 54], and the *Eucalyptus viminalis* dieback [55]. Our continuous failure to slow down climate changes will likely lead to higher climate variability in the near future [56], and with this comes the difficulty of predicting the outcome of plant–microbiome interactions and, thus, the potential spread of complex plant diseases.

### (Re)emerging plant diseases

Major shifts in climate can foster the emergence of new threats to the plant holobiont health, which include pathogens that under new conducive microbiome and climatic conditions can emerge or re-emerge as serious threats to crops and forests, while they were previously irrelevant or manageable [14, 57]. This is the case, for example, of *Phytophthora nicotianae* on tomato and potato plants or *Diplocarpon coronariae* on apple trees, as reviewed in Miller et al. [14]. Climatic shifts can be strong drivers for the re-emergence of plant pathogens as serious threats to crops and forests. For example, *Corynespora cassiicola* was first considered a minor pathogen of soybean, but it has been recently reported to cause 10% to 42% yield losses in North and South America [58]. Similarly, *Phytopythium vexans* has been recently reported as an agent of root rots in raspberry [59], apple and peach [60], and kiwifruit vines [61]. Global changes and intensive agricultural practices can also push the expansion of the host range of plant pathogens and an increased susceptibility of the host plant [18], ultimately resulting in the emergence of diseases on a wider set of hosts. For example, the generalist pathogens *Sclerotinia sclerotiorum* has been recently reported to cause rots at the shoot base of kiwifruit vines in Italy [62] and lentil plants in India [63]. Similarly, several species within the genus *Phytophtora* have been recently reported in new hosts worldwide, including western hemlock in England [64], the orchid *Ansellia africana* in South Africa [65], and *Metasequoia glyptostroboides* in China [66]. In addition to expanding the host range, pathogens can expand their geographical range, as increases in environmental temperature can allow a pathogen to survive in new locations, and global trades can facilitate the introduction of pathogens in new areas. This is the case, for example, of *Xylella fastidiosa*, which was never reported outside the Americas before being associated with the Olive Quick Decline Syndrome in Italy in 2013 [67]. Climate change can restructure the ecological context in which holobiont and holobiont–pathogen interactions occur, enabling minor, geographically restricted, or host-limited pathogens to transition into emergent threats through combined effects on pathogen virulence, host susceptibility, and interactions within the microbiome.

## Microbiome-based solutions

Several studies show that plants alter the diversity and structure of their microbiome in response to biotic stresses, including herbivores [68–73] and pathogens [74–78]. These studies leverage the ‘cry-for-help’ model to link stress-induced changes in the plant microbiome as a consequence of the alteration in the plant metabolome, suggesting that such changes are beneficial to plants as they help to recruit beneficial microorganisms [79]. While a great deal of research supports the idea that plant-induced changes in microbiome composition can alleviate stressors or negatively influence pathogens, only a few studies directly link (a) changes in root exudates following a stressor to (b) changes in plant microbiomes as a consequence of changes in root exudates to (c) beneficial consequences to plants. For example, infection of tea plants by the agent of the gray blight (*Pseudopestalotiopsis camelliae-sinensis*) causes increases in the exudation of phenolic acids and flavonoids, which contribute to the recruitment of beneficial microorganisms with disease suppressive effects [80]. Similarly, individuals of *A. thaliana* grown for several cycles on soil conditioned with the pathogen *Pseudomonas syringae* pv. *tomato* showed higher exudation of amino acids and long-chain organic acids compared to the control group, which helped enrich soils with disease-suppressive microorganisms [81]. In another model system, foliar infection of the plant *Panax notoginseng* by the fungal pathogen *Alternaria panax* resulted in an increased exudation of long-chain organic acids, sugars, and amino acids from roots, which in turn showed antagonistic activity against the pathogen and fostered the recruitment of beneficial microorganisms [82]. In a detailed study, Stringlis *et al.* [83] describe how the antimicrobial coumarin scopoletin, exuded by roots in *A. thaliana*, selectively inhibits the fungal pathogens *Fusarium oxysporum* and *Verticillium dahliae* while fostering growth-promoting and resistance-inducing rhizobacteria. Unfortunately, the literature lacks examples on the host-driven microbiome modulation within the holobiont to reduce the impact of complex syndromes, but the examples above suggest that we can leverage these mechanisms to manage and engineer the plant microbiome to guarantee holobiont health. Approaches can include enriching plants and/or soil with beneficial microbes, enhancing the mechanisms behind their recruitment, providing them with a more suitable environment where they can thrive, or teaming up better combinations of hosts and microbiomes to overcome such challenges. This array of approaches provides flexibility in tailoring microbiome-based solutions to different environmental contexts and under climatic variability, as discussed below.

One common example of microbe-based solutions for plant health is soil inoculation with individual bacterial species or mixes of different strains of the same species, such as biocontrol agents, biofertilizers, or biostimulants [84]. These biocontrol agents might act directly on the pathogen, or their inoculation can result in changes in the soil microbiome, leading to disease suppression [85, 86]. Although several commercial products have been developed, only a few of them lead to consistent positive results [87]. For example, inocula of AM fungi are popular biofertilizers used in agriculture, but the commercialized products often do not lead to the promised outcomes [88, 89]. An additional step to engineer microbiomes can be taken by assembling small communities of microorganisms (~5–15 strains) to influence plant growth and health to a level that individual strains are not able to achieve. These communities of microorganisms, commonly known as microbial consortia or Synthetic Communities (SynComs), are showing great promise in novel plant protection strategies [90–92], and have a higher growth-promoting activity than single strains [93]. For example, SynComs have been successfully assembled to contrast the fungal pathogen *F. oxysporum* in tomato [94], peanut [95], and watermelon [96]. Similarly, SynComs have been successful in providing protection from *Botrytis cinerea* in tomatoes [97], from *Verticillium* wilt in cotton [98], from the root-knot nematode in cucumbers [99], and in contrasting the negative effects of the apple replant disease [100]. Interestingly, a combination of bacterial phages has been successfully used to reduce the incidence of *Ralstonia solanacearum* on tomato plants [101]. The incremental beneficial action of combining the potential of multiple microorganisms might be a key to engineering microbiomes for holobiont health. SynComs give us the opportunity to tailor their composition to work in specific environments, with the possibility of adjusting their assembly to maintain their performance under climatic variability.

While SynComs shows great promise, we also learned that individual strategies are usually not successful in the long term [102], and it is important to integrate their use in a wider context of microbiome management. Indeed, novel strategies are being deployed to enhance crop production by managing the plant microbiome structure and function, including, for example, soil microbiome transplantation. In this case, susceptible plant genotypes or disease-conducive soils can be inoculated with microbial communities from resistant plant genotypes or disease-suppressive soil, and the resulting structure of the soil- or host-associated microbiome can protect plants from pathogens. For example, the microbiota associated with eggplant genotypes resistant to *R. solanacearum* has been inoculated in a susceptible tomato genotype, resulting in up to 47% reduction in disease incidence [103]. Similarly, soil conducive to *Rhizoctonia solani* and *F. oxysporum* inoculated with a disease suppressive soil was able to reduce disease severity [104]. In addition to microbiome transplant, the direct application of plant root exudates involved in beneficial plant–microbiome interactions in field conditions has shown promising results in altering the soil microbiome to promote plant growth [105] and could be the basis for new innovative and sustainable agricultural crop protection strategies. Indeed, a previous study focusing on microbiome management compared crops using single or multiple genotypes of the same plant species and found that genotype mixture altered the soil metabolome and microbiome, contributing to the suppression of the pathogen *F. oxysporum* [106]. It is, thus, clear that multiple strategies can be deployed at the same time, which can improve the success of microbiome management programs when predicting climatic variability, and the efficiency of individual strategies in a changing climate, becomes a challenge. Indeed, in the context of new global challenges a one-size-fits-all approach might not be suitable for being deployed in a variety of field conditions. Thus, one way to address global changes might be via tailoring microbiome-based solutions locally rather than attempting to provide a global solution. While this will require additional efforts in design, production, and regulation of SynComs and overall microbiome management programs, it might be the key to improve the resilience of our crops to global changes with minimal impacts on the environment.

If we look at plants as holobionts, we begin to understand that their health and productivity are inextricably linked to the composition and function of their microbiomes. However, plant breeding and plant–microbiome interactions are two aspects of applied plant biology that are often treated individually. Crop breeding is usually informed by mapping the association between loci in the genome and phenotypic traits (e.g. yield, height, color), and this can be applied to microbiomes as well. Indeed, previous research identified plant loci associated with variation in microbiome composition in maize [107, 108], sorghum [109], *A. thaliana* [110, 111], barley [112], tomato [113], switchgrass [114], rice [115], citrus [116], and fonio millet [117]. Similarly, plant traits can be mapped towards microbiome composition and function. This approach enabled the identification of microbial biomarkers associated with key agronomic traits in foxtail millet [118]. Combining both genome-wide association studies (GWAS; host) and microbiome-wide association studies (MWAS; microbiome), we can thus identify (i) the plant genes associated with microbiome structure/function and (ii) the microbial taxa or genes that are associated with a plant phenotype. This combined approach can help us to breed crops and their microbiome at the same time, which, in combination with SynComs and other microbiome management techniques, can open the doors for a sustainable and microbiome-based revolution for cropping that is tailored to local environmental conditions.

It is, thus, clear that we are at a major turning point, where the development of microbiome-based solutions is moving fast steps towards the replacement of several chemical inputs used to sustain crop growth and health [84, 119, 120]. The examples highlighted above can be the solution to counteract complex diseases, (re)emerging diseases, and dysbiotic disorders by engineering plant microbiomes resilient to global changes and repressive to the invasion of plant pathogens (Fig. 1). It is important to note that we are still at an early stage of developing microbiome-based solutions in agriculture. The next steps will need to focus on bringing those innovations to the field, which might be facilitated by the adoption of new tools and frameworks to understand the complexity of the interactions within the holobiont and between the holobiont and the environment.

While results are encouraging additional research, moving from the bench to the field will require additional testing beyond model plants, together with wider investigations on the persistence of microbiome-based solutions after each application (i.e. for how long a SynCom application generates beneficial effects?), their stability after production or use (e.g. shelf-life and storage conditions for SynComs, or stability of co-bred plant–microbiome combinations), and how resilient they are to agricultural practices (e.g. tillage, fertilization) and to fluctuations of the abiotic environment (e.g. pH, nutrients, water). Microbiome-based solutions might not come without risks, and this aspect is often not discussed. National regulations often require strict testing on genomic and functional traits (e.g. production of toxins/metabolites, virulence factors, antibiotic resistance genes, plasmids/mobile elements), together with plant, animal, and environmental toxicological studies. However, the short- and long-term effects of the interactions of novel microbial strains within the holobiont, under the influence of global changes, might lead to unforeseen negative consequences for the host and the environment. As we propose above, this risk can be reduced by tailoring microbiome-based solution to the local context, working on enhancing the local microbiome rather than sourcing strains from other locations. It is thus essential for policymakers, industry, academy, and farmers to work together to outline how the use of SynComs will be regulated, in particular considering how stakeholders and consumers might react to their availability and use.

## Big data, large models, new frameworks

Merging microbial ecology, plant pathology, and many of the agricultural sciences comes with technical and theoretical challenges. For example, studying the holobiont requires generating data on both the host (genome, transcriptome, proteome), all its microbial associates (metagenome, metatranscriptome, metaproteome), and the metabolome that both use for generating interactions. This is why frameworks like holo-omics [121] can be helpful in guiding the understanding of the plant holobiont and digging deeper into the mechanisms that regulate it in steady-state (control) conditions and when under threat from pathogenic organisms. On the other hand, holo-omics require generating high amounts of non-targeted, multi-dimensional, and multi-layered data, which, in other words, means characterizing all the genomes, transcriptomes, proteomes, and metabolomes within the holobiont and reassembling a mechanistic model to understand links and interactions between the different components within and between organisms. This not only requires generating and managing large, high-dimensional and heterogeneous datasets and high computational efforts and expertise, but also requires new tools and theoretical basis to re-frame plant pathology in the new era of holobiont biology.

Artificial intelligence is gaining a big momentum thanks to the spread of large language models (e.g. GPT, Gemini, DeepSeek), and it can be pivotal in understanding the holobiont and harnessing microbiome-based solutions. Indeed, one of the tools available within the domain of artificial intelligence is machine learning (ML), which can be used to learn patterns from wide and complex datasets and generate predictions when providing the model with a new input. ML already found applications in plant disease detection, using data from remote sensing technologies like hyperspectral imaging, which can detect early stress responses in plants and identify specific pathogens [122, 123]. ML can also be used to screen host plants for resistance [124] and microorganisms for their pathogenic ability [125]. In microbiome science, ML has proven to be a useful resource for classifying microbiome features (e.g. diversity, composition), studying the complex interactions between different components of the microbiome, and assisting multi-omics data integration [126, 127]. The ability to learn microbiome features opens the doors for new strategies for early disease detection. Indeed, while metagenomics tools have been providing new ways to detect plant pathogens using eDNA by matching short reads to known references, those methods are not able to detect emerging pathogens, complex syndromes with unknown agents, and cases of dysbiosis, and are strongly biased towards model organisms. ML, on the other hand, does not rely exclusively on known information, but it can learn patterns from training datasets and use these models to predict the occurrence of plant disease on the basis of the whole microbiome structure. Thus, ML is a potential tool for predicting the occurrence of plant pathogens from a variety of samples, also in scenarios where current tools cannot predict the interactions within the holobiont, such as the forecasted climatic changes. ML has been successfully used to detect the presence of plant pathogens using microbiome data, for example, by predicting Huanglongbing infection in citrus plants [128, 129] and detecting the agents of *Fusarium* and *Verticillium* wilt diseases using soil microbiome data [130, 131]. In addition, ML has been successful in detecting the presence of plant pathogens without being trained on a known reference genome [125]. In the same study, the authors suggested the potential use of ML models trained on a specific host-pathogen system to be used on detecting pathogens in different host-pathogen systems [132]. Besides the detection of known threats to plant health using microbiome data, ML is a powerful tool for detecting microbiomes that are conducive to complex syndromes. Indeed, ML and microbiome data have also been successfully employed to identify biomarkers for Ginseng Rusty Roots [133], a complex syndrome of ginseng plants with still unclear etiology [134]. ML is also a powerful tool for understanding disease suppressiveness and guiding the assembly of disease-suppressive SynComs. Through this approach, Zhang *et al.* [135] identified microbial biomarkers from disease suppressive soils and used this information to guide the assembly of a SynCom able to reduce the impact of *P. syringae* pv. *tomato* DC3000 in *A. thaliana*. Within a similar host-pathogen system, ML improved the ability to screen and assemble a wide range of potential SynComs for their ability to suppress a plant pathogen [136]. The ability of ML models to detect unknown threats, and to guide the assembly of SynComs, is thus a powerful tool to guide crop protection under high climatic variability, as novel challenges to holobiont health (e.g. novel syndromes, pathogen host range expansion or (re)emergence) can be promptly detected before spreading beyond control, and SynComs can be quickly re-designed to improve their performance under a varying climate. However, it is important to note that previous research has been mostly focused on model pathosystems, and further work is needed to transfer their use in field conditions.

These new approaches and results are changing the way we look at holobiont–pathogen interactions and the way we usually approach plant pathology. For example, the disease triangle is commonly used in plant pathology to convey the message that a disease is the outcome of the three-way interaction between the pathogen, the host plant, and the environment. While it has been subjected to criticisms and revisions, its simple representation of the factors influencing a disease is what makes the disease triangle a great abstraction of reality [137]. On the other hand, the emerging threats to plant health that go beyond the ‘one pathogen–one disease’ paradigm, the new insights into holobiont biology, and the results showing promise in microbiome management to protect our crops and forests challenge the classical concept of disease triangle. Similarly, Koch's postulates, another staple of plant pathology, are commonly used to identify the agent of disease and require the constant association of a pathogen with the disease, together with the ability to isolate the pathogen *in vitro* and re-inoculating it on healthy plants, on which the pathogen needs to cause the disease, and it needs to be re-isolated *in vitro*. As for the disease triangle, Koch’s postulates are difficult to fulfill in cases such as complex syndromes or dysbiosis [138]. These concepts can be further questioned if we accept the challenge of defining a plant pathogen [139], considering that the distinction between pathogenic and beneficial microorganisms is often unclear, and beneficial microorganisms can also be detrimental to plants [140–142], or plant pathogens can become beneficial to plants [143].

**Figure 2:**
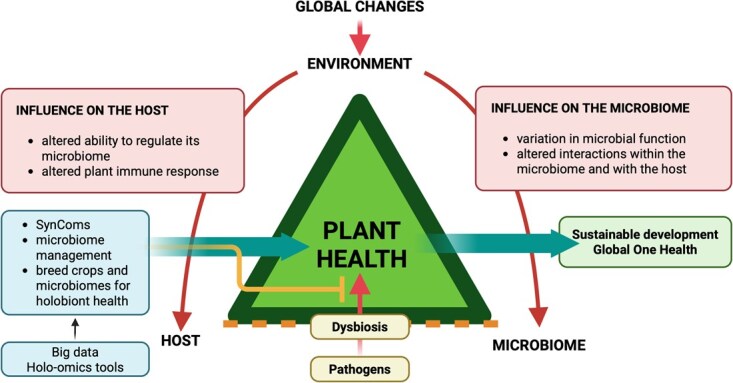
Environmental variation due to global changes can influence both the host and microbiome components of the holobiont. In the host, climate changes can cause a loss of the ability to regulate the microbiome and to recruit beneficial microorganisms, which, together with an altered plant immune response, can lead to suboptimal interactions with the microbiome. On the microbiome side, climatic changes can alter the microbial function (e.g. reduce redundancy or complementarity) and the interactions within the microbiome and with the host plant. Ultimately, all these effects can impair host–microbiome interactions and cause dysbiosis within the holobiont. This can give the opportunity to new, emerging, and re-emerging pathogens to cause detrimental effects to plant health, and cause novel diseases that do not fit inside the ‘one pathogen–one disease’ framework. However, the characterization of healthy and diseased holobiont with the holo-omics tools, together with the power of artificial intelligence, can help us to deploy microbiome-based solutions (e.g. SynComs, microbiome management, breeding crops and microbiomes) to restore the host–microbiome interactions within the holobiont, producing positive effects on plant health contributing to the sustainable development and promoting global One Health. Created in https://BioRender.com

How do we move forward to integrate these two important concepts for plant pathology into a holobiont biology perspective? Two recent papers highlight possible strategies [137, 138]. According to Leveau [137], we can move from the concept of the disease triangle to the one of the health triangle, which shows the interactions between the host, microbiome, and environment as major factors in guaranteeing plant health. This alternative also fits the holobiont framework, seeing pathogens as an integral part of the host microbiome, that their ability to cause disease is under the influence of both the host and other members of the microbial community, and that a dysbiotic microbiome can be a driver of disease. Also, moving away from the ‘disease’ triangle towards the ‘health’ triangle fits better with the integration of agricultural practices and products into planetary One Health [137, 144]. Similarly, as suggested by Bonello [138], plant health challenges that go beyond the ‘one pathogen–one disease’ paradigm can be treated with modern meta-omics or holo-omics tools to identify molecular signatures for the disease when classical Koch’s postulates cannot be applied. Together, these updated approaches can provide a holistic framework for the mechanisms driving the detrimental host–microbiome interactions and their negative influence on the holobiont, and together with new holo-omics tools and ML, can help to deploy microbiome-based solutions to improve plant and planetary health (Fig. 2).

## Conclusions

Researchers worldwide are calling for microbial stewardship and microbial solutions against the threat of global changes [19, 145–148], and understanding plant health through the lens of holobiont biology represents a transformative step in addressing the dual challenges of global changes and sustainable agriculture. Unlike traditional frameworks, holobiont biology can support the management of complex syndromes and emerging diseases. This holistic perspective enables innovative, microbiome-based strategies that can enhance plant resilience, mitigate pathogen threats, and reduce dependency on environmentally harmful agrochemicals. With tools, such as holo-omics and ML, we can now uncover complex interactions within plant holobionts, enabling predictive and proactive solutions on a scale previously unimaginable.

These solutions align directly with global imperatives, such as the United Nations Sustainable Development Goals [149], by contributing to food security, biodiversity conservation, and climate-resilient agricultural practices. To fully unlock the potential of microbiome-based strategies, we must prioritize scaling these innovations, integrating them into policy, and fostering collaboration across disciplines. Researchers and stakeholders must work together to advance this field. Practical next steps include scaling microbiome-based solutions through industry partnerships, conducting large-scale field trials in diverse climates, and integrating microbiome management into global policy agendas for sustainable production. These efforts will ensure that the potential of microbiome science translates into real-world impacts, driving progress toward resilient and sustainable agricultural systems.

As agriculture increasingly faces the pressures of climate variability and resource scarcity, embracing holobiont biology offers a path to reimagine crop systems that are both productive and sustainable. This paradigm not only strengthens our understanding of plant health but also aligns with the broader ‘One Health’ framework, addressing the interconnected well-being of humans, plants, animals, and ecosystems. By fostering this new understanding, we can ensure thriving agriculture in the face of global changes, ensuring a resilient and sustainable future.
